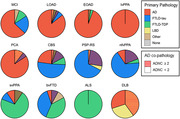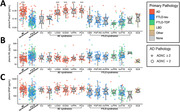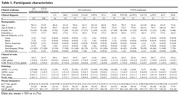# Diagnostic Performance of Plasma P‐tau217, NfL, and GFAP for Predicting Alzheimer’s Disease Neuropathology Across Diverse Neurodegenerative Syndromes

**DOI:** 10.1002/alz.090596

**Published:** 2025-01-09

**Authors:** Hanna Cho, Mark E. Sanderson‐Cimino, Jonathan Tablante, Fattin Wekselman, Lea T. Grinberg, Hilary W. Heuer, Argentina Lario Lago, Peter A. Ljubenkov, Julio C. Rojas, William W. Seeley, Salvatore Spina, Adam M. Staffaroni, Gallen Triana‐Baltzer, Hartmuth C. Kolb, Renaud La Joie, Gil D. Rabinovici, Adam L. Boxer, Lawren VandeVrede

**Affiliations:** ^1^ Memory and Aging Center, UCSF Weill Institute for Neurosciences, University of California, San Francisco, San Francisco, CA USA; ^2^ Gangnam Severance Hospital, Yonsei University College of Medicine, Seoul Korea, Republic of (South); ^3^ Department of Pathology, University of California San Francisco, San Francisco, CA USA; ^4^ Neuroscience Biomarkers, Janssen Research & Development, LLC, San Diego, CA USA; ^5^ Memory and Aging Center, University of California, San Francisco, San Francisco, CA USA

## Abstract

**Background:**

In the era of disease‐modifying treatments for Alzheimer's disease (AD), accurate detection of underlying AD pathology is critical. Blood‐based biomarkers for AD are increasingly available, but their diagnostic performance is not well‐understood across the spectrum of neurodegenerative disease, especially when AD presents as co‐pathology in non‐AD syndromes. We investigated the diagnostic performance of three plasma biomarkers (phosphorylated tau 217 [p‐tau217], neurofilament light chain [NfL], and glial fibrillary acidic protein [GFAP]) to detect AD, confirmed by autopsy, across 12 clinical neurodegenerative syndromes with various underlying etiologies.

**Methods:**

A total of 349 individuals with ante‐mortem blood samples and post‐mortem neuropathological examination were included at UCSF between August 2008 and March 2021. The participants were clinically diagnosed with syndromes related to AD, frontotemporal lobar degeneration (FTLD), or dementia with Lewy bodies (DLB) or were cognitively unimpaired (CU) controls. Quantification of p‐tau217, NfL, and GFAP was performed using SiMoA assay by Janssen. Clinically relevant AD neuropathology was defined as intermediate or high AD neuropathological changes (ADNC ≥ 2).

**Results:**

Significant AD neuropathology was observed in 88% (110/125) of AD‐related syndromes and 23% (45/198) of FTLD‐related syndromes. Plasma p‐tau217 levels were higher on average in AD‐related syndromes (0.28±0.16pg/mL) compared to FTLD‐related syndromes (0.10±0.09pg/mL, p<0.001). All AD‐related syndromes, except for mild cognitive impairment, showed significantly increased p‐tau217 levels compared to CU (0.10±0.08pg/mL, p<0.001). No significant differences were observed in NfL and GFAP levels when directly comparing AD‐related (31±28pg/mL[NfL], 267±131pg/mL[GFAP]) and FTLD‐related syndromes (50±50pg/mL[NfL], 201±155pg/mL[GFAP]) or either group with CU (32±14pg/mL[NfL], 235±67pg/mL[GFAP]). Plasma p‐tau217 demonstrated excellent diagnostic accuracy in detecting AD neuropathology, achieving an AUC of 0.975[0.946‐1.000] in AD‐related syndromes (0.973 sensitivity and 0.800 specificity) and 0.888[0.831‐0.944] in FTLD‐related syndromes (0.467 sensitivity and 0.961 specificity). In contrast, NfL and GFAP showed lower diagnostic performance in AD‐related syndromes (AUC=0.857[0.748‐0.966] and AUC=0.809[0.661‐0.956], respectively) and even less accuracy in FTLD‐related syndromes (AUC=0.740[0.662‐0.740] and AUC=0.755[0.679‐0.831], respectively).

**Conclusion:**

AD neuropathology was prevalent across diverse neurodegeneration syndromes, both as primary and co‐pathology. Our findings suggest that plasma p‐tau217 could be a useful tool for identification of AD neuropathology in the presence of non‐AD neurodegenerative syndromes, potentially for identifying populations for investigation of effects of AD treatments.